# Modeling the appearance and progression of cognitive impairment

**DOI:** 10.1192/j.eurpsy.2021.381

**Published:** 2021-08-13

**Authors:** B. Mainguy, L. Mehl-Madrona, D. Ruben

**Affiliations:** 1 Education Division, Coyote Institute - Canada, Ottawa, Canada; 2 Medical Arts And Humanities Program, University of Maine, Orono, United States of America; 3 Research Division, Coyote Institute, Tucson, United States of America

**Keywords:** computer simulation modeling, cognitive impairment, prediction, major neurocognitive disorder

## Abstract

**Introduction:**

It remains difficult to predict which individuals will develop cognitive impairment and progress to major neurocognitive disorders. Prevention studies suffer from the long time frames and the manner in which this topic does not lend itself to randomized, double-blinded controlled trials.

**Objectives:**

We aimed to construct a computer simulation model that would accurate portray the time course for a series of individuals to develop cognitive impairment and to progress to major neurocognitive disorder.

**Methods:**

We built a computer simulation model that incorporated the role of exercise, genetic load, age, quality of diet, presence of diabetes and level of hemoglobin A1C, ongoing levels of cognitive stimulation, presence or absence of micronutrients, presence or absence of other co-morbidities, an overall general health index, levels of smoking and other substance use, and family history. We modeled the life course of 10 individuals, adjusting parameters to make correct predictions for all 10 people. Then we entered the data from another 10 people to determine how accurate the model would be with ten new individuals for whom it had not been developed.

**Results:**

We defined success as a prediction of onset within 10% of the actual date and a prediction of the slope of the trend within 20%. We had 7 successes. We were able to engage 6 of the 10 in interacting with the model to change health behaviors.

**Conclusions:**

Computer simulation modeling may provide an opportunity to study the long-term effects of health behaviors and to engage people in interacting with the program to change behavior.

**Disclosure:**

No significant relationships.

